# High Pressure Anesthesia

**Published:** 1906-03-15

**Authors:** S. M. Weaver


### HIGH PRESSURE ANESTHESIA.

BY S. M. WEAVER, D.D.S.

Requirements.—First. There was a good deal of conjecture at first as to what effects this method would have on the pulp, but I am pleased to state that after about two years' continued use, I have the first case yet to report of the slightest disturbance whatever. The first cases on which the instrument was used, have been in my office frequently and I always test them to see if they are normally sensitive, and cannot detect the slightest difference. I have even used the same pit to obtund a tooth for filling three different cavities at intervals of six months, and that tooth is to-day perfectly normal as far as sensation is concerned, as any tooth in the mouth, proving in my judgment, the harmlessness in its use, and if space permitted, could cite many similar cases, but remember, it is hard to make any method Fool Proof and no matter what the subject is, serious thought, caution and common sense should be exercised.

Second. After a certain amount of study, experimentation and practice, any cavity can be thoroughly anesthetized by the high pressure method. The trouble is, most operators try some one of the different makes of instruments and, without any previous experience to familiarize themselves with the instrument at hand, try to obtund a difficult case, and because they fail, throw the instrument into a drawer and condemn the method, instead of hiring a small boy to kick him for trying to do something which he had not given the proper amount of thought. Who would expect to make a perfect inlay the first time they read an article on the subject of inlays, or insert a *perfect* gold filling the first time he attempted the operation?

Third. *The time consumed* in operating by the high pressure of course, varies with the case and make of instrument used; for example, an elderly patient with a receded pulp would take more time than a child with open tooth structure. On the other hand, an instrument that depends upon the strength or grip of the fingers, will not work as quickly as one where the pressure is obtained by powerful mechanical devices, as seen in several makes on the market. The average time of operation I would say, is about two minutes, which would be very conservative, while a great many only require about one-half minute. From the previous statements, it will readily be seen that the high pressure has no competitor as far as time is concerned.

Fourth. One sitting is all that is necessary. The length of anesthesia can be controlled to a certain extent by the skill of the operator, depending upon the age of patient, position of cavity, kind of solution—that is whether pure cocain is used or a combination of adrenalin and cocain —and again as to the instrument used.

Fifth. After considerable practice, you will be able to judge the extent you wish to anesthetize the tooth to be operated upon. For instance, you are to insert a large gold filling and you want the anesthetic to work until the polishing is completed, to obviate the pain caused from heating the gold by friction, you would inject more anesthetic than you would if the case was to be an amalgam where you wanted the anesthetic to last just while cutting. Mind you, I do not claim you can do this exactly, but by practice you will be surprised how expert you will become in this one feature.

Sixth. It must perfectly obtund any and all cases.

The high pressure comes nearest the ideal for all cases. If a patient is immuned to cocain, which is very rare, you can readily see the uselessness of trying to make cocain do the work, but in two years' use of the method, I have experienced but two cases like above mentioned, and both of these have later caused me to think that it was their imagination entirely. Teeth having congested pulps and those having pulp stones are much slower to act, owing to the impaired circulation, but if one keeps trying, the result can be easily accomplished.

In a short time, the dentists over the country will begin to realize that the one thing that they have been waiting for for years is embodied in the principles of high pressure, and only time and experience will bring out its great usefulness that is sure to come, and those men that are standing back and criticising it *untruly*, from a theoretical standpoint, will soon realize their mistake and join with their more fortunate brothers who have enjoyed its benefits from the start.

I used cataphoresis for several years, but when high pressure came, my cataphoric apparatus has never been called upon, for the same results are obtained in a few seconds that used to take, I might say, hours. But I am something like the farmer who had an old horse past usefulness. When his neighbors asked him why he did not kill it, the farmer answered: "I am keeping him for the good he has done in the past." And so it is with my cataphoric outfit.

The time is not far off when every dentist will consider a high pressure instrument as necessary a part of his equipment as he does the dental engine to-day.

Patients are already demanding its use, in sections where it has become generally known. If the public could be educated as to the benefit of the high pressure method, every dentist would be compelled to make one a part of his outfit.

If the dentists used high pressure for only two things, namely: *Removal of pulps* and *obtunding abutments* for bridges, they would relieve the patient of two most disagreeable operations. At present there is a great wave of refrom going over the country, condemning the use of Arsenous acid for devitalizing. Now, why "try and plough with a wooden stick" by applying cocain on cotton and then trying to force it through the tubuli by pressing soft rubber into the cavity when by drilling a pit with a one-half bur, the high pressure does the work in a few seconds? After drilling until the pulp is reached, by a second injection of a very few seconds, the pulp can be removed from any tooth without the slightest pain and in much less time than it takes to tell it. This eliminates completely the argument for arsenic.

Never fill a root canal at the same sitting after the removal of a pulp with cocain, particularly when adrenalin is combined with the solution. First, because of the secondary hemorrhage, and second, the tissue surrounding the apical space being anesthetized renders it impossible to work with the same degree of accuracy as when this space is sensitive, especially when forcing in the root filling.

Take, for example, a bridge that is to be swung from the second upper molar to the first bicuspid and, as in many cases, the molar is perfectly sound, we all know that to dress it down *perfectly*, without a great deal of pain, *has* been an *impossibility*, and the result is seen every day, by the worst kind of ill-fitted crowns, causing the condemnation of bridge work instead of the condemnation of the operator, and really it was not the operator's fault, but the physical impossibility of the patient to endure the pain. It is not necessary now.

The end of one of the cusps is ground off, exposing the dentin. This can be done without a particle of pain, if good, true, carborundum stones are used with plenty of hot water. After this is accomplished, drill with a one-half bur directly towards the pulp until sensitive dentin is reached Then apply the high pressure for about twenty seconds, which is generally sufficient in a healthy tooth. You will now be able to grind as much as you like without any pain to the patient, perfoıming your work thoroughly and much easier, saying nothing of the time saved. After thoroughly trimming the abutment, if the pit has been ground out, make another in the same place and dismiss the patient.

We all know how sensitive these abutments are after they have been ground down for two or three days, as is generally necessary while constructing large bridges. Ordinarily the patient suffers severe pain during the fitting on account of the hyper-sensitiveness, and when air is applied to dry the abutment, followed by the action of the phosphoric acid in the cement, the pain is terrific. This operation generally taxes the limit of endurance in both patient and operator, but let us look at it from a more humanitarian standpoint. The tooth has been dressed down without pain, the pit was drilled while the tooth was anesthetized, and all there is to do is to insert the needle of the high pressure in the pit already made and inject until desensitized, which is readily ascertained by having your assistant blow a little cold air on the tooth, and the patient will do the rest. By this method, a minimum of pain has been caused, and the rest of the operation, such as fitting, drying the abutment and cementing in place is accomplished without a particle of pain. Operators who have experienced this pleasure and satisfaction, could not be made to discard it at any cost and your patient will go out of the office pleased and gratified instead of being a physical wreck, and dreading the time when he will have to come again. If there is any other method that will do this same work with the same degree of success, I will be thankful to learn of it. The profession has been imposed upon by the flooding of the market with cheap, poorly made instruments, and many operators are condemning the method instead of the instrument. There are several good ones on the market and some operators are more successful with one than the other according to their individual ideas, but I am sorry to say, a good share of our profession want something *cheap*, no matter what the result is.

The essentials of a good instrument are as follows:

Must operate easily.

Must be finely adjusted.

Must be one handed.

Must cause a minimum pressure against the tooth.

Must have a direct line of force.

Must have a constant or live pressure.

Must be quickly filled.

Must be durable and so constructed that it can be cleaned daily without taking to pieces.

In conclusion, I wish to predict that within a short time high pressure will be as universally used to obtund teeth as gold is for a filling material.

